# Supplementary figures and images for: Automated, stress-free, and precise measurement of songbird weight in neuroscience experiments
Source: PLoS One. 2026 Jan 12;21(1):e0339848. doi: 10.1371/journal.pone.0339848 (PMC12795351; doi:10.1371/journal.pone.0339848)

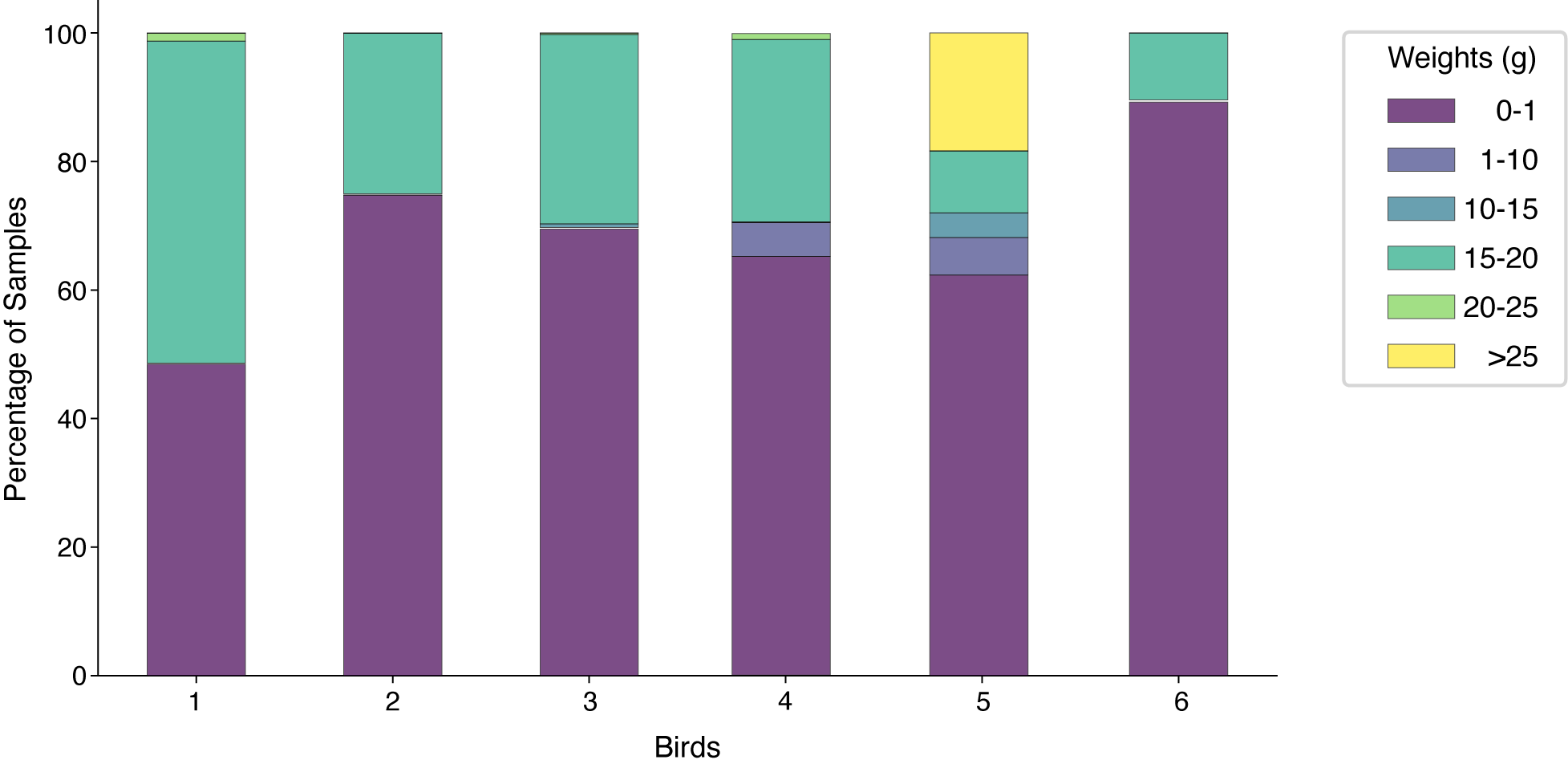

Supplement: S1 Fig — Stacked bar plots illustrate the distribution of perch-scale weight measurements across different weight ranges for each bird. In all cases, the majority of data points fall in the 0–1 g range, reflecting off-scale baseline moments when the bird was not perched. These baseline values dominate the raw dataset and highlight the need for filtering before extracting meaningful weight information. Beyond these off-scale readings, most valid measurements fall within the expected canary weight range (15−−25g), consistent with true body weights. However, additional “noise” measurements are present outside this range, caused by factors such as transient signal fluctuations from wing flaps near the perch (0−−5g), unstable or partial perching positions that yield misleading but relatively stable values (e.g., 5−−15g), and rare impact artifacts producing transient, unrealistically high weights (>30g). In one device (’Bird 5’), loss of calibration resulted in a persistent bias toward excessively high values, further illustrating the need to monitor the calibration of these devices, especially within the first days upon setup. Together, these distributions emphasize the rationale for applying cutoffs to exclude off-scale values and outliers, ensuring that analyses focus on reliable, stable measurements within the biological range of the birds. (TIFF) [file pone.0339848.s001.tif]

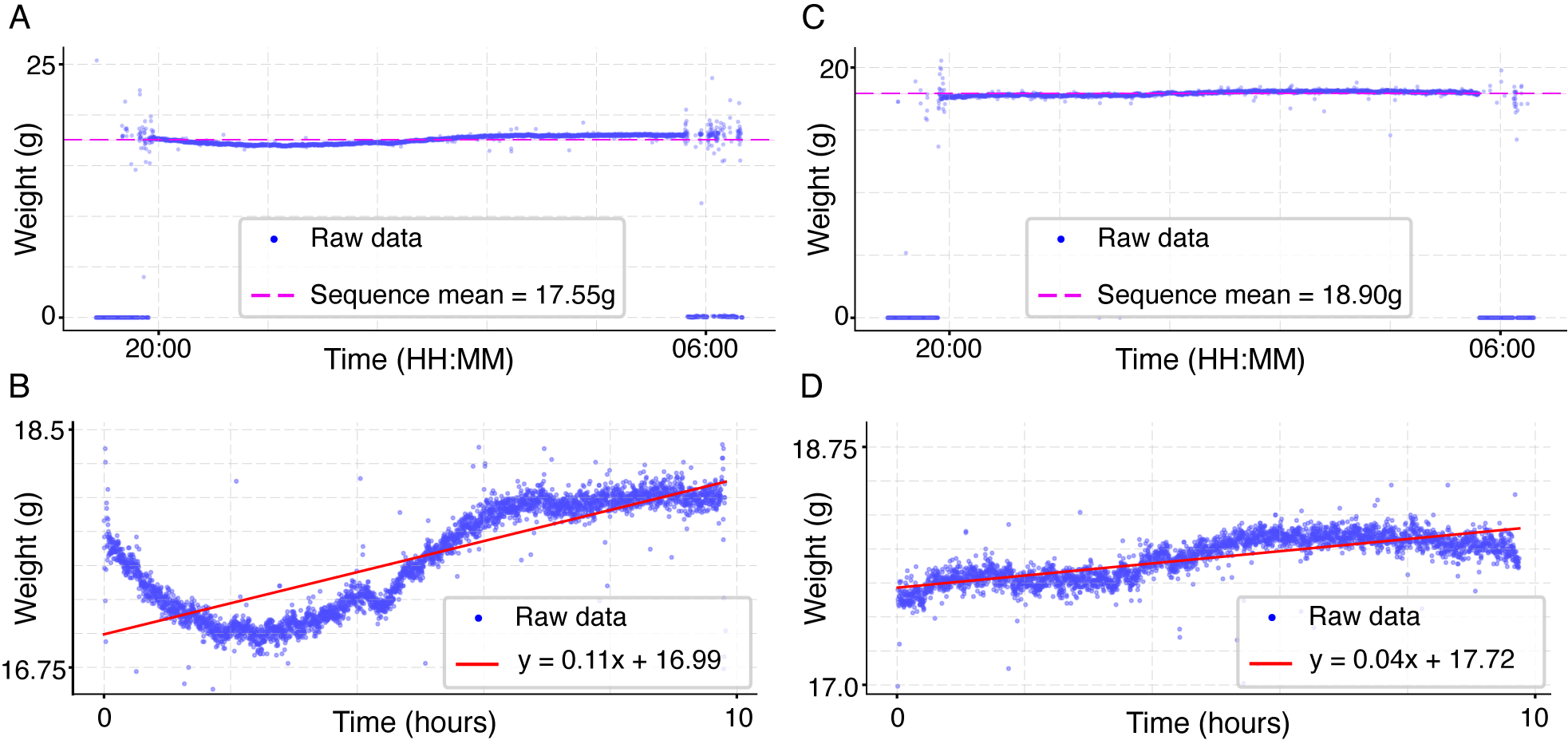

Supplement: S2 Fig — In these two occasions, the perch-scale does not indicate weight loss trends, as opposed to the overnight weight loss trends captured in the other 22 sessions. Panels A-D show a closer look into these two overnight sessions, where panels A and B relate to one sequence, and panels C and D relate to a second sequence, both from the same perch-scale and bird, on consecutive nights. It is apparent that the linear trend is somewhat unstable, especially in the case shown in panels A and B, unlike the 22 other sessions where the linear trend was stable (as shown in main Fig.4A). This could suggest that these two cases shown here fail to capture weight loss as a result of device malfunction rather than a real trend. These are brought up here to show a potential example of how noise or miscalibration affects the perch-scale data. Occurrences like this should be closely monitored to evoke recalibration of the perch-scale. (TIFF) [file pone.0339848.s002.tif]

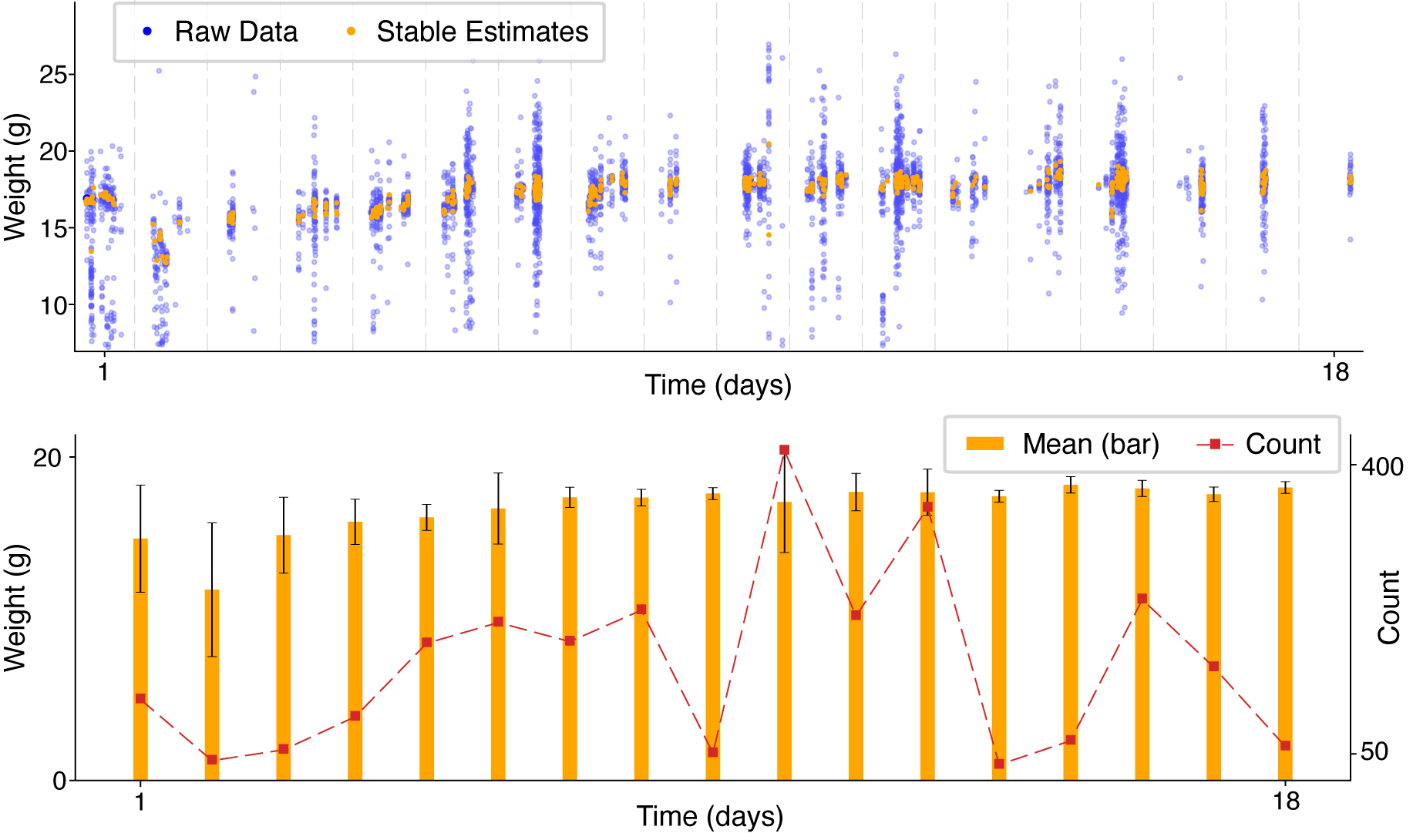

Supplement: S3 Fig — Scatter plots (top) show raw measurements (blue) and stable estimates (orange) collected continuously across multiple consecutive days for a bird in the pilot experiment. The corresponding bar plots (bottom) summarize daily mean stable weights (bars, left axis) alongside the number of stable estimates contributing to each mean (red line, right axis). Although no manual weights were collected for direct comparison in this pilot, the data demonstrate that the perch-scale device can reliably generate stable daily weight estimates over extended periods of time, with hundreds of stable measurements per day. These results provide additional evidence for the long-term robustness and reliability of the system, complementing the main manuscript figures where manual weights were available for validation. (TIFF) [file pone.0339848.s003.tif]

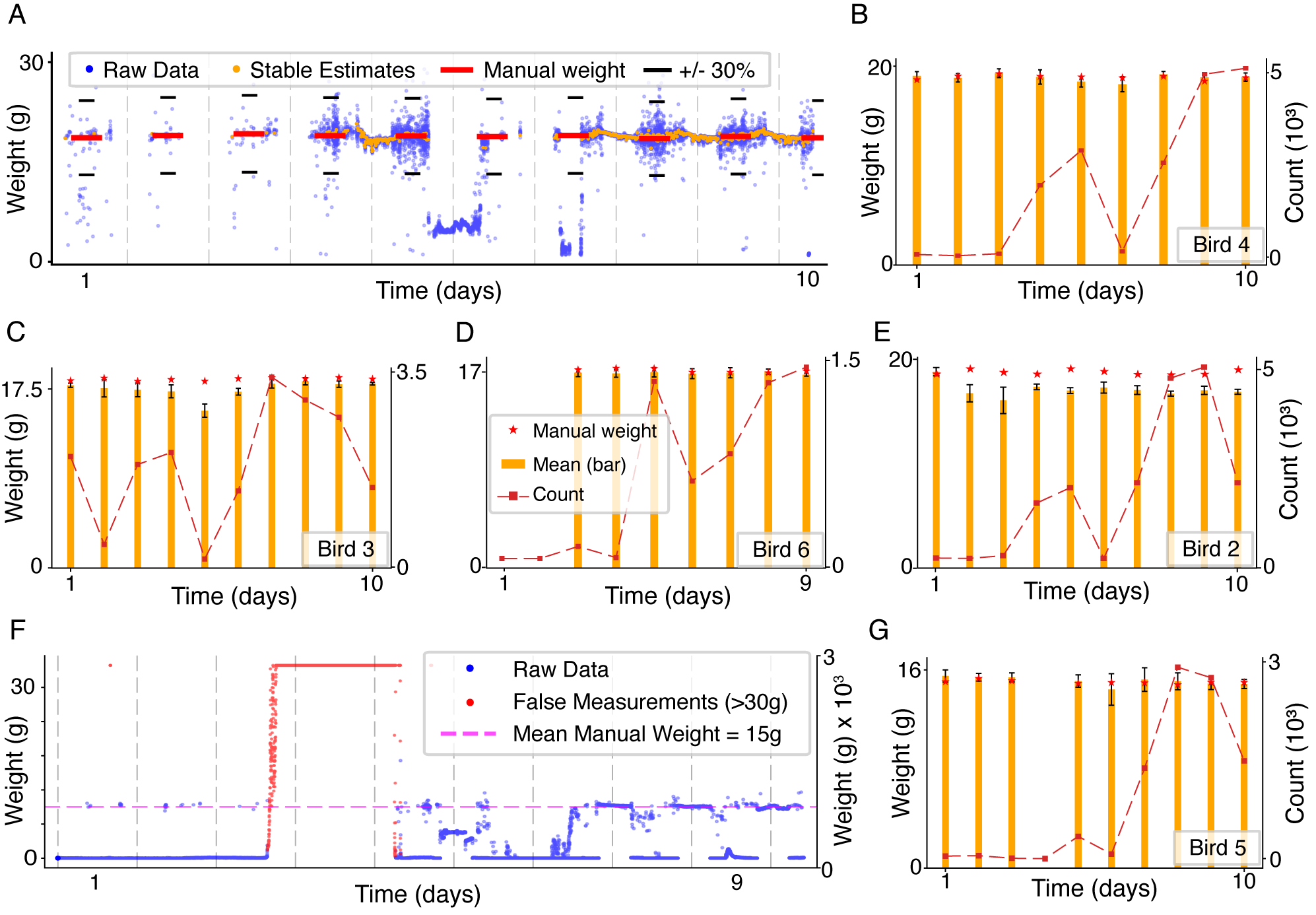

Supplement: S4 Fig — To continue the results shown in main Fig.5 of the manuscript, the longitudinal results of the remaining five birds monitored in the main group are shown here. (A) Similar to Fig.5A, an example of continuous perch-scale measurements from a representative bird, across ten days. Raw weight data are shown as blue scatter points, while stable weight estimates (orange points) align closely with the manual daily weight measurements (red lines). Black lines are added above and below the daily manual weight, representing the ±30% range used as a cutoff threshold when analyzing the daily estimates. (B) Summary of daily stable weight estimates for the same bird shows the mean of all stable estimates within each day (bars, left axis) with accompanying counts of stable estimates per day (line, right axis). (C-E) Summary of daily stable weight estimates from three other birds. The estimates summary for these birds, as well as the bird shown in panels A and B, is relatively consistent and closely matches the daily manual weights. Note here that the the data in panel E (bird 2) is consistently biased. This bird’s data was removed from the summary analysis due to this bias, and this serves as an example of the need to monitor the calibration. (F) Scatter plot of raw data (blue points) from one perch-scale that lost calibration (bird 5) and was re-calibrated at the beginning of day 5. Red scatter points represent false weight measurements as a result of this miscalibration. These red points are scaled down by 50 to match the scale of the blue dots (right axis). The perch-scale data previous to the point of miscalibration (end of day 3) are scarce, although a few accurate measurements closely align with the mean of daily manually measured weight (dashed magenta line). The weight measurements following the recalibration process (day 5 and on) are relatively stable and closely align with the mean of daily manually measured weights. (G) Summary of stable estimates per day for bird 5. (T [file pone.0339848.s004.tif]

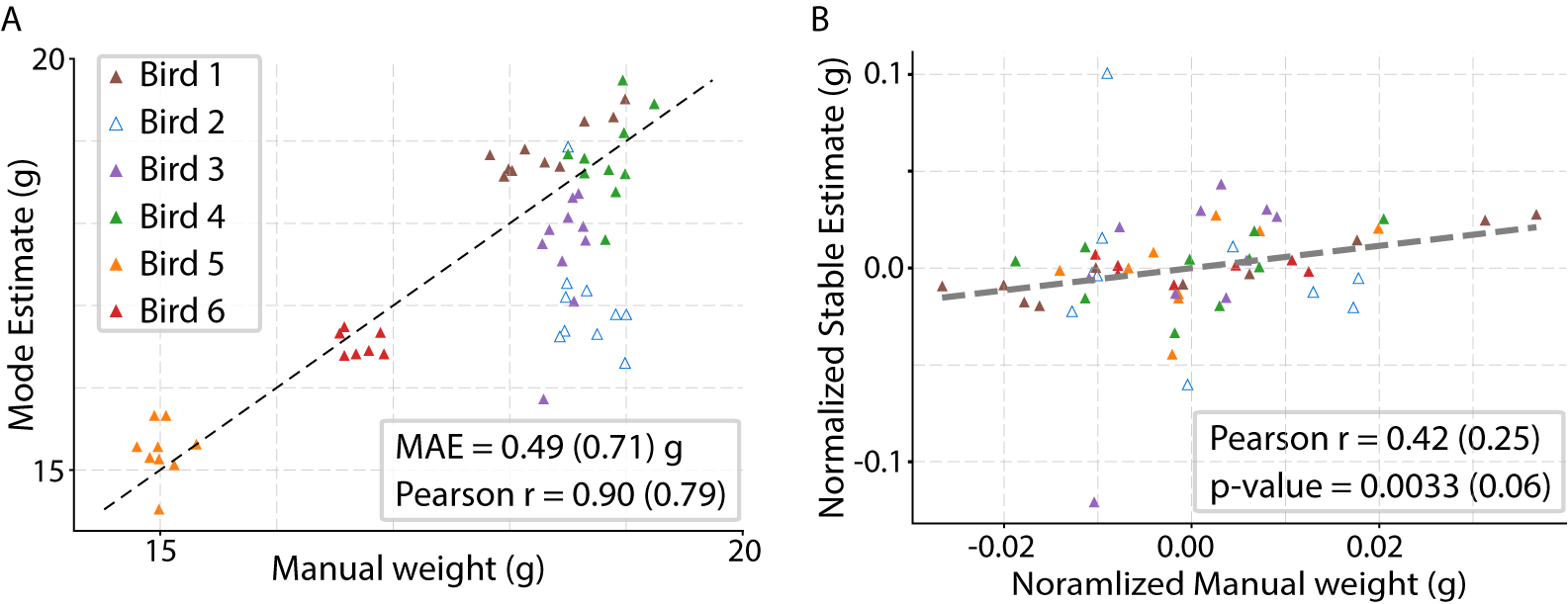

Supplement: S5 Fig — Daily weight estimates derived from the perch-scale are compared with manual weights collected once per day. Each point represents data from one day, with the x-axis showing the manually measured weight and the y-axis showing the perch-scale estimate. Colors correspond to individual birds, and open blue triangles indicate an outlier bird with biased measurements; removing this bird improved accuracy. (A) Mode-based estimates: perch-scale daily mode values plotted against manual weights. Accuracy metrics are shown with and without the outlier bird (values in parentheses indicate before removal). Removing the outlier improved the mean absolute error (MAE) from 0.71g to 0.49g and the Pearson correlation from r = 0.79 to r = 0.90. (B) Stable-estimate values normalized within each bird, enabling comparison of fractional daily changes independent of baseline weight differences. The linear regression fit (dashed line) demonstrates a significant positive association with manual weights (r = 0.42, p = 0.0033). This panel highlights that perch-scale data not only captures absolute weights but also tracks day-to-day fluctuations in body weight consistent with manual measurements, reinforcing its reliability for monitoring subtle longitudinal changes. (TIFF) [file pone.0339848.s005.tif]
