# Supplementary material for: Automated, stress-free, and precise measurement of songbird weight in neuroscience experiments
Source: PLoS One. 2026 Jan 12;21(1):e0339848. doi: 10.1371/journal.pone.0339848 (PMC12795351; doi:10.1371/journal.pone.0339848)
Supplement: Attachment — Submitted filename: BonnehEtAl_ResponseLetter.pdf [file pone.0339848.s006.pdf]

Response to referees and editorial suggestions for our manuscript entitled *"Automated, stress-free, and precise measurement of songbird weight in neuroscience experiments"*

We thank the referees for their careful reading and additional guidance. Below, please find a detailed response to the reviewers' concerns and comments. *Reviewers' comments are shown in italics*, with our detailed responses following each comment; changes in the paper are shown in blue in the manuscript file.

1 Reviewer #1

1.1 Major Concerns

- *The authors housed five canaries in one cage and the sixth canary in another cage. How did they achieve weight measurements for each animal? Are all the data in Figures 2 and 3 from the singly housed bird? If so, the authors should clarify that they only measured the weight of one animal, rather than six. If not, the authors should explain how they identified which animal was measured in each data point.*

  The perch-scale device we developed monitors the weight of individually-housed, often tethered, birds in neurophysiology experiments. A single control unit can connect to and manage up to eight such devices, allowing simultaneous monitoring of multiple birds. Following the reviewer's comment, we emphasized this design choice in the manuscript; (1) In the last paragraph of the introduction: "...connecting multiple devices, each monitoring the weight of one bird, to a centralized control system that handles setup and configuration.". (2) In the Materials and Methods - Overview: "...Multiple birds can be monitored...as each bird is assigned to an individual perch-scale...". (3) In the Experimental Procedures - Birds: "...housed individually in cages equipped with one perch-scale device...". When presenting the data in Figures 2,3 (Now Figures 3,5), we stressed that the data is from two different singly-housed birds. Following further comments (below), we also include data from six more birds that were monitored in either of the two experiments we conducted, in Figure 5 and as Supporting Information Figures S1-S5.

- *The raw measurements varied in a range of 10-14 grams. Although the authors have shown that the scale was accurate for measuring objects, there is no ground truth measurement of the birds' weights. The authors should compare their measurements with measurements of restrained birds, which is not difficult to do.*

  We thank the reviewer for this comment. To address this concern we conducted two additional experiments. In the first, we used several inert objects to test the calibration of the weighing device in a larger range (5-40 grams). We include the result as a new figure (Figure 2). In the second experiment, we tracked the weight of six new birds over a period of ten days.

We monitored the weight using individual perch-scale devices while also manually measuring their true weight once per day using a cloth restraint and a standard scale device. We include this additional data in Figures 3-5 as well as in Supporting Information Figures S1-S5

- *Figure 2C showed that the distribution of data is skewed. The authors chose to use the mean, rather than the mode, as the weight estimate but did not explain the rationale. There is a good reason to consider the mode as a better estimate: When animals stay still, the measured data are centralized in a narrow range and are more accurate, so it is unclear whether the estimate is biased.*

  We agree with the reviewer and refined our analysis rationale, which is now better explained in the new Methods - Data Analysis and Results sections. First, to establish our rationale for filtering the data, we added Figure 3 - an example of the raw perch-scale data during daytime activity in one day. The figure shows that: (1) most of the data points are baseline, unperched moments, and (2) there are moments of spurious data points due to irregular perching and other noise factors (these points are further discussed in the mentioned sections, as well as in the Supporting Information Figure S1). To address these notions, we suggest here to: (a) remove baseline, near-zero data points before observing the data and calculating metrics such as the mode, and (b) apply a cutoff of ±30% of the bird's ground truth weight before carrying further analyses, to minimize false detection of weight changes due to noise and irregular perching. Then, to obtain the best estimate for the bird's weight from the perch-scale data and compare it with the daily ground truth measurements, we explicitly define two methods. The first is, as the reviewer suggested, the mode of daily weight measurements, and the second is based on perched periods with minimal variations, which we refer to as "stable perch-scale estimates". The rationale for choosing the ±30% cutoff and the stable perch-scale estimates is further explained in the Methods - Data Analysis section of the revised manuscript. We hope that the addition of this new information better supports our analysis approach.

1.2 Minor Concerns

- *The authors should explain in more detail the housing conditions in the method section. For example, what was the lighting condition in the aviary?*

  To address this concern, we revised the Methods - Birds section. The revised section now reads: "...birds were housed individually in cages equipped with one perch-scale device each, and placed inside an acoustic chamber (TRA Acoustics, custom-made with size $65 * 50 * 50cm$)... Lighting conditions inside the acoustic chambers were similar among all birds, mimicking the outside photoperiod by turning the artificial lights on and off according to a controlled light cycle.".

- *The authors measured the over-night weight changes in one bird three times but only presented one result. The authors should present the other two results to verify the consistency of the effect.*

  Indeed, this phenomenon was captured several times in the first experiment, and in the new experiment we conducted for this revision, it was captured numerous times, with one bird sleeping on the perch-scale every night. The new Figure 4 shows one such example in detail (Panel A) and a collective plot displaying additional occurrences (22 in total) with a global fit line that indicates an average weight loss of $0.07g$ per hour.

2 Reviewer # 2

This manuscript addresses a relevant and important aspect of animal welfare and data collection in neuroscience experiments involving birds. The authors have developed an automated weighing system designed to minimize stress during weight measurements, offering substantial advantages over traditional manual methods. Overall, I find the work relevant, useful, and detailed. However, I have some minor suggestions for improvement and clarification:

- *Authors should cite and discuss relevant prior work, example in original mail. I also recommend a detailed literature search, it took me 1 minute to find this paper, there are possibly more.*

  Following the reviewer's comment, we cite three more very relevant works that demonstrate automated weighing systems for aviaries, from which we also derived the term *Perch-Scale*:

  - Poole, A. (1982). A scale for weighing birds at habitual perches. Journal of Field Ornithology, 53(4), 409-414.
  - Claffy, J. The Small Bird Perch Scale: a Remote Weighing Apparatus. AFA Watchbird, 29(1), 77-80. (The reviewer's reference)
  - Vézina, F., Charlebois, D., & Thomas, D. W. (2001). An automated system for the measurement of mass and identification of birds at perches. Journal of Field Ornithology, 72(2), 211-220.

  Citing these references allowed us to make clear the contribution of our work. Briefly, these works present a similar concept of the scale device and they suggest the name 'perch-scale'. But, they lack the longevity, the parallel control of multiple devices, and fit for use with tethered birds that we suggest here. We revised the introduction of our manuscript to emphasize the existing literature and these problems in existing approaches that our development addresses. The relevant paragraph now reads: "Automated perch-based weighing systems have been available and in use as early as in the 1980s - including the *Small Bird Perch-Scale* for small passerines in zoos (Claffy et al., 2002), field applications for large birds such as ospreys in their habitat (Poole et al., 1982), and later fully automated solutions combining perches with electronic identification for monitoring captive and wild birds (Vezina et al., 2001). These devices were primarily developed for general health monitoring, ecological studies, or husbandry, rather than the specialized needs of neuroscience experiments. Our perch-scale system is designed to meet those particular needs by: 1) minimizing the risk of cable entanglement to safely accommodate birds tethered to data acquisition devices, 2) collecting reliable, long-term weight data continuously and from multiple birds in parallel, and 3) connecting multiple devices, each monitoring the weight of one bird, to a centralized control system that handles setup and configuration.". We also revised the discussion part to make our contribution clear. The relevant paragraph now reads: "This weighing system builds on established perch-based weighing systems successfully used in both captive and wild contexts (Claffy et al., 2002, Poole et al., 1982, Vezina et al., 2001). Our contributions are mainly adapting the design to meet the specific needs of neuroscience studies on tethered birds in acoustic chambers; The system delivers reliable, long-term weight monitoring, ensures the safety of birds tethered to sensitive data acquisition setups, and supports extended periods of uninterrupted, high-quality recordings of both weight and neurophysiological data. Furthermore, a single centralized control unit manages multiple devices, streamlining data collection, configuration, and calibration.".

- *The authors state that their system can monitor multiple birds simultaneously, but it appears that each bird must be housed in a separate cage. This important detail should be explicitly clarified. Since housing birds together would require individual bird identification, it would be beneficial if the authors discussed how their multi-bird system provides a distinct advantage compared to simply replicating a single-bird system multiple times.*

  To follow this concern, we revised the Introduction and Materials and Methods sections. In both sections, we emphasize that each bird is housed in a single cage with an individual perch-scale, as one system (Raspberry Pi + Arduino) can control and gather data from up to 8 devices in parallel. The revised Introduction section now reads: "...connecting multiple devices, each monitoring the weight of one bird, to a centralized control system that handles setup and configuration....We demonstrate here that our system allows reliable tracking of individual birds' weight...". In the Materials and Methods section, we further emphasize that each bird must be singly-housed. The revised section now reads: "Each *Scale System* can connect up to eight individual perch-scales per microcontroller...This way, multiple birds can be monitored for their weight at the same time, as each bird is assigned to an individual perch-scale placed inside its cage" (Overview section), "...birds were housed individually in cages equipped with one perch-scale device each, and placed inside an acoustic chamber (TRA Acoustics, custom-made with size $65*50*50cm$)..." (Experimental Procedures - Birds section), "The recording of weight data from all birds was done using a Python script that runs on a Raspberry Pi minicomputer (the control unit) which receives a list of eight data points, each referring to one weight reading from a different perch-scale, every second. This data is organized to match each of the weights to the relevant bird..." (Data Collection section). While our system is not capable of monitoring the weight of multiple birds with one weighing device, it is mostly built to fit with birds tethered to an acquisition device in neurophysiology experiments, in which they are usually housed alone. Nevertheless, our system's ability to handle multiple such devices in one place is one of the main contributions.

- *All this threshold choosing and outlier removal might be unnecessary. What authors do seems to be more complicated than what is necessary. Couldn't the authors measure the median which is robust to outliers instead of the mean? So, choosing that 10 g threshold would be unnecessary. Finally, authors should simply show all measurements rather than to arbitrarily cut off 'off-scale' values (<10 g) and outliers (>30 g). If they absolutely need that 10 g threshold, then they need to provide guidelines or a "recipe" for adopters of their method on how to choose that cutoff in practice. In summary, I recommend reducing the need for complicated threshold-based data cleaning.*

  We thank the reviewer for this comment as it helps us better clarify this topic. Our new perch-scale design is mainly derived from the need to safely accommodate birds tethered to acquisition devices with cables. To avoid cable entanglement, the device has to be located as closest to the floor as possible. This design can evoke irregular perching, which leads to noisy, unreliable data that might be less prevalent in other designs, in which the perch-scale is attached to the cage walls. To address this issue, we came up with this analysis rationale that includes data filtering. Hence, our main aim here is to provide he readers with all of the relevant information on the perch-scale data. To do that, we added two figures to the revised manuscript; (1) The new Figure 3 shows the raw perch-scale data of one representative bird during daytime of one day. It emphasizes some of the common characteristics of this data, including the proportion of perched to unperched measurements as well as how noise, possibly due to irregular perching, can affect the analysis. (2) Supporting Information Figure

S1, referenced in the methods section, shows that the majority of weight measurements are baseline, unperched moments and thus simply calculating the mode or median does not produce valuable information without any filtering. It also shows that in some occasions, the proportion of noise and irregular perching to reliable, perched measurements, can be too big to be ignored. On top of that, we explain in the Materials and Methods - Data Analysis section how we analyzed the perch-scale data, to extract the most reliable bird weight estimations for comparison with the manually measured weights (the new experiment we conducted following this review included acquiring daily weight measurements for each bird manually).

To follow the reviewer's request, we simplified our "recipe" for cutoff thresholds to be more direct. It involves applying a cutoff threshold of ±30% of the bird's ground truth weight, excluding all measurements not within this range. The rationale for these values is mentioned in the new "Filtering the Data" subsection, which now reads: "This threshold accommodates the detection of substantial welfare red flag (defined as a drop of 20% or more from baseline weight (Morton, D. B., & Griffiths, P. H. (1985)))while minimizing false deviations. An additional 10% was added to include potential measurement bias from the device".

Following this cutoff phase, we used two methods to estimate the birds' daily weight to compare with the manual measurements, as mentioned in the new Data Analysis - Overview section, which now reads: "...Our analysis workflow first involved filtering...then generating two types of daily weight estimates: (1) the *daily mode*, a fast, frequency-based estimate, and (2) the *stable perch-scale estimate*, a more selective approach for identifying perched periods with minimal variation.".

We hope the added information result in a more descriptive manuscript. Future users of this device can consider following the methods described here, or they can learn the characteristics of the perch-scale data and specify them for their experimental conditions. All of our data and analysis scripts are available via external links mentioned in the manuscript.

- *The sentence stating that "two birds were excluded for not producing enough reliable data" is somewhat vague. The clearer statement from the discussion section seems to be that "from two of them (out of six) we were not able to gather substantial weight data". The authors should explicitly indicate that these two birds presumably did not sit frequently or steadily enough on the perch to generate sufficient data. Ideally, authors quantify the fraction of time in which reliable measurements are possible.*

These vague statements were removed in the revised manuscript. We conducted a new experiment in which we removed alternative perching options other than the perch-scale. In this experiment, the birds stood on the perch-scale 56 out of 60 days (ten days times six birds), compared to the lower reliability in the pilot experiment where birds had multiple perching options. We mention this point in the Discussion section, which now reads: "In our experiment, birds had no alternative perching options other than the perch-scale, except for the food and water containers, mounted on lower cage walls. This encouraged frequent use of the perch-scale and ensured high-quality data throughout the ten-day experiment, with some birds sleeping on the perch-scale overnight every night. In contrast, in the earlier pilot group... approximately one-third of birds rarely used the perch-scale, preventing weight estimation. Based on these observations, we recommend minimizing alternative perching options in future studies employing this system.". We also added clear exclusion criteria to the Methods - Overview section. The revised section now reads: "Days (24-hour periods) that did not yield stable perch-scale estimates were excluded from the analysis, as they did not provide sufficient and reliable data for daily weight assessment.". This defines the four days out of 60

that were excluded, as mentioned in the Results section: "...based on 56 successfully-obtained daily estimates out of 60 (the missing four include days when the perch-scale did not produce *Stable Perch-scale Estimates* within the filtered range at all...".

- *The term 'Reliable Weight Measurement' appears more as a descriptive term rather than a clearly defined data characteristic. In addition to presenting examples of unprocessed measurements, authors should illustrate the criteria and procedures used to deem measurements "reliable."*

  To follow the reviewer's comment, we refined the term *Reliable Weight Measurement* and renamed it to *Stable Perch-scale Estimate.* We added a subsection to Methods - Data Analysis section, which now reads: "To obtain a more robust estimate of the bird's weight, we also calculated the *stable perch-scale estimate.* This method uses a rolling window of 10 samples (approximately 10 seconds) applied to the filtered dataset. For each window, we computed the standard deviation (SD) and retained only those with $SD \leq 9\%$ of the bird's manual weight. Each qualifying window's mean value was recorded as one stable estimate. A daily weight estimate was then computed as the mean of all stable estimates within that day. Beyond producing a single daily value, this approach allows visualization of fine-scale fluctuations throughout the day, provided the bird frequently uses the perch. The choice of a 10-sample window and a 9% SD threshold was determined via grid-search hyperparameter tuning.". To illustrate this data characteristic, we also added Figure 3 - an example of the raw perch-scale data during daytime activity of one day. The figure shows that: (1) most of the data points are baseline, unperched moments, (2) there are moments of spurious data points due to irregular perching and other noise factors (these points are further discussed in the mentioned sections, as well as in the Supporting Information Figure S1), and (3) how the *stable perch-scale estimates* accurately capture the true weight of the bird, after filtering the raw data. These notions provide a basis for our rationale for filtering the data and why we chose to use these *stable perch-scale estimates* as another metric for estimating bird's weight accurately.

- *I recommend showing measurements on inanimate objects first to determine system noise. Only then show measurements of animals at night where they move less, then during the day, when they move, and then when they were tethers. Z-scoring the values would allow making these measurements comparable.*

  We agree with the reviewer. Accordingly, we reorganized the manuscript. The order of results in the revised manuscript is now: (1) Validation of the perch-scale (inanimate objects). (2) Raw data characteristics and filtering approach (daytime example from one bird). (3) Overnight measurement weight loss. (4) Longitudinal across days. (5) Summary (normalized).

  First, we show the perch-scale performance on inanimate objects, with additional objects so that we now cover weights of $5 - 40g$ (Fig. 2 in the revised manuscript). Then, we show one representative example of daytime-only data in order to display general characteristics of the raw, unfiltered data, as a basis for our analysis rationale (Fig. 3). Following those, we move on to the main part of the results - in which we chose to display overnight (Fig. 4), then longitudinal (Fig. 5, day and night together), including the tethered bird (Panels D-E in Fig. 5). Lastly, a summary of the analysis results, including normalizing the daily comparisons for each bird to their fractional changes is mentioned in the results section and shown in Supporting Information Figure S5.

- *The sentence "the canary stood on the scale for long enough" implies a duration, but the provided measure appears to be a count. Clarify whether N represents the average number of reliable measurements per day or another specific measure*

  We agree with the reviewer. To address this comment, as well as the comment about the Reliable Estimate, we first changed this term to a clearer and more robust method, which we call 'Stable Perch-scale Estimates' in the revised manuscript. This helped us improve the definition of what data is "reliable", as these 'Stable Estimates' refer to perching moments when the perch-scale measurements within a ten-second window had relatively low variability (see reference to the revised Methods - Data Analysis - Stable Perch-scale Estimates). Then, we only used data that passed the filtering and the 'Stable Estimates' approach when we calculated daily weight estimates to compare with the manual measurements. Therefore, all weight estimates that were used can be defined by these metrics, and days when "the birds did not stand on the perch for long enough" are now defined as days without such 'Stable Estimates'. We use the parameter N here to indicate the prevalence of these 'Stable Estimates' within each day, with a mean count of $\bar{N} = 1859.5$ (ranging between $6 - 5123$). Thus, the method for calculating the 'Stable Estimates' is the criteria (as days without such estimates are excluded), and the parameter N provides more information about this metric. The relevant text in the revised Results section now reads: "Across all six birds in the main group (see section Birds), we obtained on average $\bar{N} = 1859.5$ (range 6–5123) stable estimates per day, with a typical fluctuation of $0.43g$ around the daily mean, based on 56 successfully-obtained daily estimates out of 60 (the missing four include days when the perch-scale did not produce *Stable Perch-scale Estimates* within the filtered range at all - see Methods-Data Analysis section)."

- *The authors should reconsider the phrasing "natural weight loss," replacing it simply with "weight loss," unless "natural" is explicitly and meaningfully defined in the manuscript.*

  Agreed. This sentence was rephrased to *physiologically relevant* for this reason as well as to be a better fit to the new structure of the overnight section in the results. The relevant section now reads: "...These results validate the perch-scale's sensitivity to physiologically relevant weight fluctuations, highlighting its potential for detecting subtle health changes in birds.".

2.1 Concern raised in the editor's notes

How well would this system work for smaller birds if the variance is not proportional and there is some absolute noise?

This is a valid point raised by the editor. As the research in our domain of neurophysiological experiments focuses on songbirds like canaries and zebra finches, we developed this perch-scale to fit such birds. We do show in Figure 2 of the manuscript the accuracy and validity of our device on innate objects ranging between 5 and 40 grams. The results indicate no changes in variance across different weights within this range. The perch-scale was also shown to be accurate by $\approx \pm 0.5g$ when monitoring the weight of birds for long periods. Although we did not try monitoring smaller birds, this accuracy could enable capturing changes of up to 10% of bodyweight even in birds that weigh 5 grams.

To conclude, although we only tested the device with innate objects in the range of 5 to 40 grams, as well as with birds that weigh 15 to 20 grams, it could have the potential to work with lighter or heavier birds. Other researchers that opt to use this device for in such cases, can adopt our design and code for their specific use.
